# Wine Yeast Peroxiredoxin *TSA1* Plays a Role in Growth, Stress Response and Trehalose Metabolism in Biomass Propagation

**DOI:** 10.3390/microorganisms8101537

**Published:** 2020-10-06

**Authors:** Víctor Garrigós, Cecilia Picazo, Emilia Matallana, Agustín Aranda

**Affiliations:** 1 Institute for Integrative Systems Biology, I2SysBio, University of Valencia-CSIC, 7, 46980 Paterna, Spain; victor.garrigos@uv.es (V.G.); picazoc@chalmers.se (C.P.); emilia.matallana@uv.es (E.M.); 2 Department of Biology and Biological Engineering, Chalmers University, S-41296 Gothenburg, Sweden

**Keywords:** *Saccharomyces cerevisiae*, wine, biomass, oxidative stress, peroxiredoxins, Tsa1

## Abstract

Peroxiredoxins are a family of peroxide-degrading enzymes for challenging oxidative stress. They receive their reducing power from redox-controlling proteins called thioredoxins, and these, in turn, from thioredoxin reductase. The main cytosolic peroxiredoxin is Tsa1, a moonlighting protein that also acts as protein chaperone a redox switch controlling some metabolic events. Gene deletion of peroxiredoxins in wine yeasts indicate that *TSA1*, thioredoxins and thioredoxin reductase *TRR1* are required for normal growth in medium with glucose and sucrose as carbon sources. *TSA1* gene deletion also diminishes growth in molasses, both in flasks and bioreactors. The *TSA1* mutation brings about an expected change in redox parameters but, interestingly, it also triggers a variety of metabolic changes. It influences trehalose accumulation, lowering it in first molasses growth stages, but increasing it at the end of batch growth, when respiratory metabolism is set up. Glycogen accumulation at the entry of the stationary phase also increases in the *tsa1*Δ mutant. The mutation reduces fermentative capacity in grape juice, but the vinification profile does not significantly change. However, acetic acid and acetaldehyde production decrease when *TSA1* is absent. Hence, *TSA1* plays a role in the regulation of metabolic reactions leading to the production of such relevant enological molecules.

## 1. Introduction

Stress tolerance is a key factor for the survival and success of the microorganisms involved in biotechnological processes. The budding yeast *Saccharomyces cerevisiae* is no exception, and its ability to deal with harsh conditions is key for its proper industrial performance [1,2]. The most fitting strains to perform alcoholic grape juice fermentation to produce wine are selected according to this parameter, and they are produced as a dehydrated starter in the form of active dry yeast (ADY) [3]. That implies their growth in molasses in a scaled-up succession of bigger fermenters. Biomass proliferation increases when adopting the respiration metabolism. The transition from fermentative to respiratory metabolism causes oxidative stress, which is the main signature during that process, marked by the expression of antioxidant defenses [4]. Other relevant stress conditions are initial hyperosmotic shock and later nutrient starvation. Stress induction, particularly oxidative stress, during biomass proliferation is key for allowing successful biomass drying. That environment is harsh when osmotic stress is highly relevant, but also when oxidative damage increases [5,6]. A vital protective molecule against dehydration is the disaccharide trehalose, which is a well-known agent preventing damage to membranes and proteins under such conditions [7]. Adding chemically pure antioxidants to the molasses has been proven to be a successful way to increase cell survival and performance after drying [8]. Food-grade argan oil, rich in antioxidants such as oleic and caffeic acids, is also able to improve the dehydration process by alleviating oxidative damage such as lipid peroxidation and protein carbonylation [8,9,10]. ADY is then used as inoculum for grape juice fermentation. Vinification also has a particular signature of stresses, which goes from initial hyperosmotic shock, due to high sugar concentration, to the rise in ethanol concentration and final nutrient starvation. Stress response gene expression evolves along fermentation accordingly [11]. Eventually, all the adverse conditions lead to cell aging and death [12].

The systems of stress response are embedded in a global machinery that coordinates growth, metabolism and defense, and the nutrient signaling pathways lie at the core of this regulation [13]. Many stress insults during yeast industrial performance are metabolism-derived, such as the high ethanol concentrations produced by fermentation or the oxidative stress brought about by mitochondria due to metabolic transitions [1]. The antioxidant stress response is complex and acts at many levels [14,15]. The first line of defense involves the enzymes that eliminate reactive oxygen species (ROS), such as catalases, superoxide dismutases and peroxidases. There are two reducing systems that maintain the cell redox state and help to repair oxidative damages: one is based on glutathione and the other on thioredoxins [16]. Glutathione, γ-L-glutamyl-L-cysteinylglycine (GSH), is a protective tripeptide that constitutes the most abundant low-molecular-weight thiol of the cell. Its ability to produce an oxidized form when forming a reversible disulfide bond (GSSG), renders it a key factor for redox control. Thioredoxins are small proteins involved in thiol redox control and able to regenerate oxidized proteins by changing the redox status of their active cysteines. There are two cytosolic thioredoxins in yeasts, namely Trx1 and Trx2. They are regenerated by cytosolic thioredoxin reductase Trr1, which uses NADPH as a final donor of reducing power. In the set of proteins that rely on thioredoxins for their activity, we find thioredoxin-dependent peroxidases called peroxiredoxins [11]. Peroxiredoxins are able to reduce peroxides, specifically hydrogen peroxide in the case of Tsa1 and Tsa2, and alkylhydroperoxides for Ahp1. Those all work on the cytosol, while peroxiredoxin Dot5 functions in the nucleus and Prx1 in mitochondria. Tsa1 and Tsa2 proteins are very similar, but *TSA1* gene is highly expressed during exponential growth, while *TSA2* behaves like typical oxidative stress gene in that it is only induced by oxidative insult [17,18]. *TSA2* can complement the functions of *TSA1* when expressed by *TSA1* promoter [17].

Besides the antioxidant function, thioredoxins have been linked with metabolism control under winemaking conditions. The *trx1*Δ*trx2*Δ double mutant possesses proven diminished glycolytic activity, but increased lipid synthesis and amino acid metabolism during wine fermentation [19]. *TRR1* deletion also impacts metabolism by affecting TCA and increasing the content in proteogenic amino acids [20]. These changes can be caused by the direct regulation of nutrient signaling pathways TORC1 by Trr1 [20]. Peroxiredoxin Tsa1 is a proven multifunctional protein that acts as a redox switch to control many physiological aspects [21]. When oxidative damage is high, its own oxidation triggers multimerization and a new function as protein chaperon arises [22]. It is also associated with both ribosomes [23] and the accumulation of unfolded proteins [24].

Based on the previous analysis of the relevance of members of thioredoxin systems, such as *TRX1*/*2* thioredoxins [19] and thioredoxin reductase *TRR1* [20] in the physiology of wine yeasts, the aim of this work was to test the impact of deletion in the peroxiredoxins identified in the *S. cerevisiae* genome on yeast performance not only during imposed oxidative stress, but also in relevant industrial conditions. As the mutant on main cytosolic peroxiredoxin *TSA1* displays a growth defect under several conditions, its impact during biomass production simulations on molasses and during grape juice fermentation was tested. This showed that beside its defined role as an antioxidant, *TSA1* modulates some critical metabolic aspects, such as the accumulation of protective carbohydrates trehalose and glycogen during yeast biomass propagation, and the production of metabolites of enological interest, such as acetic acid and acetaldehyde, during grape juice fermentation.

## 2. Materials and Methods

### 2.1. Yeast Strains and Growth Media

The yeasts used in this work are described in Supplementary Table S1. Gene deletions were performed with recyclable selection marker loxP-kanMX-loxP from plasmid pUG6 [25]. Recombinase cre under the inducible *GAL* promoter to excise the *kan*MX marker was carried in plasmid Yep351-cre-cyh [26]. Yeast transformations were done by the lithium acetate method [27]. Yeasts were usually grown in rich YPD medium (1% yeast extract, 2% bactopeptone, 2% glucose). Solid plates contained 2% agar, and 20 μg/mL geneticin when required. YPS medium is as YPD, but containing 6% sucrose instead of glucose. Molasses were diluted to 60 g/L sucrose and supplemented with 7.5 g/L (NH_4_)_2_SO_4_, 3.5 g/L KH_2_PO_4_, 0.75 g/L MgSO_4_ and 10 mL/L vitamin solution. The vitamin solution contained 0.5 mg/L D-biotin, 1 mg/L calcium pantothenate and 1 mg/L thiamine hydrochloride. Synthetic molasses (SMs) were adapted from elsewhere [28] with modifications [29,30]. The SM medium contained 20 g/L sucrose (diluted molasses) for the batch fermentation phase and 100 g/L for the fed-batch process. These media were supplemented with an assimilable nitrogen source (5 g/L bactopeptone, 7.5 g/L (NH_4_)_2_SO_4_), mineral salts (1.42 g/L KH_2_PO_4_, 0.74 g/L MgSO_4_, 0.5 g/L NaCl, 0.067 g/L CaCl_2_·2H_2_O), organic acids (2904 mg/L lactic acid, 144 mg/L malic acid and 49 mg/L citric acid), with oligoelements, vitamins and anaerobic factors such as synthetic grape juice MS300 (see below). The final pH was adjusted to 4.5 with KOH. Red grape juice (Bobal variety) was a gift from Bodegas Murviedro (Requena, Spain). It was sterilized overnight with 500 μg/L dimethyl dicarbonate. Synthetic grape juice MS300 was prepared as described [31].

### 2.2. Growth Conditions

The growth curves in YPD and YPS were performed on a Varioskan Lux plate reader at 30 °C with shaking, and inoculating from a stationary culture in YPD. To measure the fermentative capacity, precultures were grown overnight on YPD, inoculated at a final concentration of 10^7^ cells/mL in synthetic must [31], and incubated at 30 °C. CO_2_ production was measured every 10 min by an Ankom RF Gas production System. Fermentative capacity was expressed as the ml of CO_2_ produced per 10^7^ cells. Growth in molasses took place in flasks cultivated at 30 °C with shaking (180 rpm), which were collected at 24 h.

The fermenter experiments were performed in a 5 L bioreactor (Applikon) with a working volume of 3 L batch SM medium. The overnight YPD precultures incubated at 30 °C with shaking (180 rpm) were used to inoculate media to an initial OD_600_ of 0.1. Temperature was 30 °C and agitation speed was 450 rpm, which were maintained throughout the process. Dissolved oxygen was followed with an O_2_ electrode and an air flux of 0.5–1.5 kg/cm^3^ was fixed. The initial pH was 4.5, which could freely vary in the batch step. During the fed-batch process, the reactor was fed with 1 L of fed-batch SM medium with 100 g/L sucrose by a peristaltic pump at the desired flow rate. pH was automatically maintained at 4.5 with 1 M KOH. Cell growth was followed by measuring OD_600_.

The vinification experiments conducted in natural grape juice were inoculated with the cells from the stationary cultures in YPD at a final concentration of 10^6^ cells/mL in filled-in conical centrifuge tubes with 30 mL of must. Incubation was done at low shaking and 25 °C. The vinification progress was followed by determining colony-forming units (by serial dilution, plating and colony counts) and sugar consumption.

### 2.3. Redox Parameters

ROS levels were measured by incubating cells in 200 μL of PBS with 10 μg/mL of DHE (dihydroethidium) for 15 min at 30 °C, and by measuring at 485/595 nm on a Varioskan Lux (ThermoFisher) plate reader [32].

Cell extracts were obtained from 100 mg of cells and were used for the lipid peroxidation, trehalose, glycogen and glutathione determinations. Trehalose and glycogen levels were determined as previously described [33,34]. Cells were resuspended in 250 mM of Na_2_CO_3_ and incubated at 95 °C for 4 h. Then, they were centrifuged at 12,000 rpm for 30 s. The supernatant was recovered and incubated overnight with 8.4 mU of commercial trehalase (Sigma) at 37 °C or the *Aspergillus niger* amyloglucosidase preparation (1.2 U/mL) at 57 °C. The released glucose was measured by the glucose-oxidase/peroxidase method. Trehalose and glycogen levels were expressed as μg of trehalose or glycogen mg of cells. Glutathione determination was carried out as previously described [35,36]. Cells were collected and resuspended in HCl 8 mM, 1.3% 5-sulphosalicylic acid. Cells were broken by vortexing at 4 °C with glass beads. Cell debris was pelleted and supernatants were used for the glutathione determinations. Total glutathione was determined using 200 µL of the supernatant, while 4 µL of 1 M 2-vinylpiridine were added to determine GSSG content. The reduced glutathione content was calculated as the difference between the total and oxidized glutathione. Glutathione levels were expressed as nmol/mg of cells.

Quantification of the lipid peroxidation in cells was measured by the reaction of thiobarbituric acid with malondialdehyde (MDA) [37], a product of fatty acid breakage, as previously described [37]. MDA content was estimated by using a standard curve with known commercial MDA concentrations. Lipid peroxidation was expressed as pmoles of MDA/mg of cells.

### 2.4. Metabolite Determinations

For the sucrose determination, samples were incubated at 30 °C for 10 min in 50 mM sodium acetate, pH 5.0 and 2.5 U invertase (Sigma). After adding 100 μL of 0.4 M K_2_HPO_4_, reactions were stopped by boiling. Samples were centrifuged and the glucose concentration was determined in supernatants by a glucose oxidase/peroxidase assay. The reducing sugars during grape juice fermentation were measured by a reaction to DNS (dinitro-3,5-salycilic acid) [38]. Ethanol, acetaldehyde, glycerol and acetic acid were measured with a commercial kit (Megazyme, Ireland) according to the manufacturer’s specifications.

## 3. Results

### 3.1. TSA1 is the Most Relevant Peroxiredoxin for Growth

**Figure 1:**
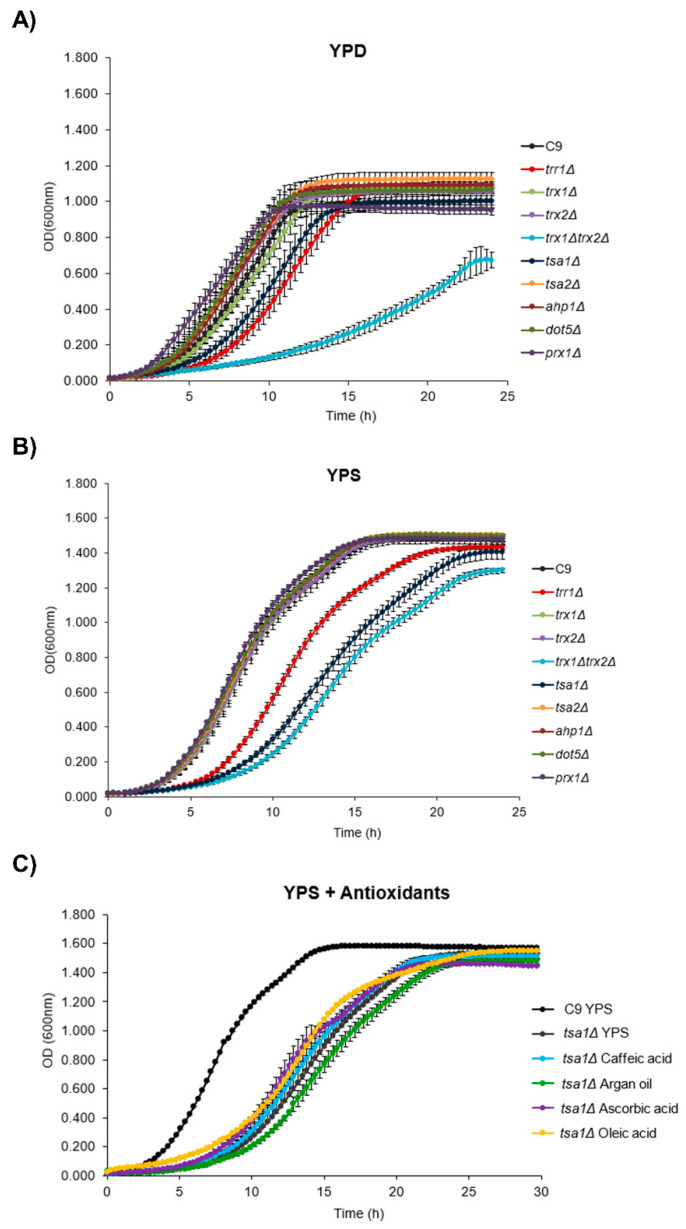
Peroxiredoxin *TSA1* deletion impacts growth. Growth curves in multi-well plate readers of mutants in several genes of the thioredoxin system in haploid wine strain C9. (**A**) Growth in YPD medium. (**B**) Growth in YPS medium (the same as YPD, but with 6% sucrose as the carbon source). (**C**) Effect on *tsa1*Δ mutant growth of adding 12 μL/mL argan oil and pure antioxidants (5 μM caffeic acid, 5 μM ascorbic acid, 6 mg/mL oleic acid) to YPS. Experiments were carried out in triplicate. The mean and standard deviation are shown.

In order to better understand the role of the thioredoxin-peroxiredoxin systems during wine yeast growth in fermentative substrates, the mutants of relevant genes were tested. Haploid wine strain C9 was used to make the process easier [39]. The mutants in all five peroxiredoxins (*TSA1*, *TSA2*, *AHP1*, *DOT5, PRX1*) were tested, together with the single mutants in cytosolic thioredoxins *TRX1* and *TRX2*, the double mutant and cytosolic thioredoxin reductase *TRR1* deletion (Figure 1). Growth in the reference rich medium YPD (Figure 1A) was impaired in the *trx1*Δ*trx2*Δ double mutant, as previously observed during grape juice fermentation [14]. The simple mutants did not show such deleterious growth, which, as expected, indicates functional redundancy. The mutations in peroxiredoxin *TSA1* and thioredoxin reductase *TRR1* showed an initial delay in growth, but both strains reached similar final cell densities to the reference strain. Mitochondrial peroxiredoxin *PRX1* deletion displayed slightly faster growth during the first hours, which suggests that exponential growth under fermentative conditions does not seem to require an active mitochondrial thioredoxin-peroxiredoxin system.

Biomass growth takes place under industrial conditions in molasses rich in sucrose, but not in glucose. As sugar beet molasses are dark in nature, a rich medium called YPS was used, which was similar to YPD, but which replaces 2% glucose with 6% sucrose, and mimics molasses (Figure 1B). Under this condition, most mutants had a negligible impact, only mutants *trx1*Δ*trx*Δ, *tsa1*Δ and *trr1*Δ, in this order, displayed delayed growth. *TSA1* was more relevant for cell proliferation in this condition than in YPD, almost as much as *TRX1/2* was, which suggests that most of the roles that are played by the thioredoxin system channel through Tsa1, or that *TSA1* has alternative roles during cell proliferation. Both growth curves indicated that peroxiredoxin *TSA1* plays a more central role than any other thioredoxin-dependent peroxidase in growth. Therefore, the following experiments focused on them.

Antioxidants have proven useful for promoting biomass grown in molasses [8]. As *TSA1* deletion is assumed to result in extra oxidative stress, growth in YPS in the presence of pure antioxidants ascorbic, caffeic and oleic acids, plus a natural source of antioxidants, argan oil, was tested (Figure 1C). None of the treatments was able to restore the growth of the *tsa1*Δ mutant to the wild type profile. Oleic acid offered some relief in the phenotype caused by the mutation, but argan oil, which is rich in oleic acid, was even more detrimental to growth. Therefore, the *TSA1* deletion phenotype may have additional functions outside the peroxidase activity, as the effect of antioxidants does not mimic the activity of peroxidases.

### 3.2. Lack of Peroxiredoxin TSA1 Diminishes Growth in Molasses

**Figure 2:**
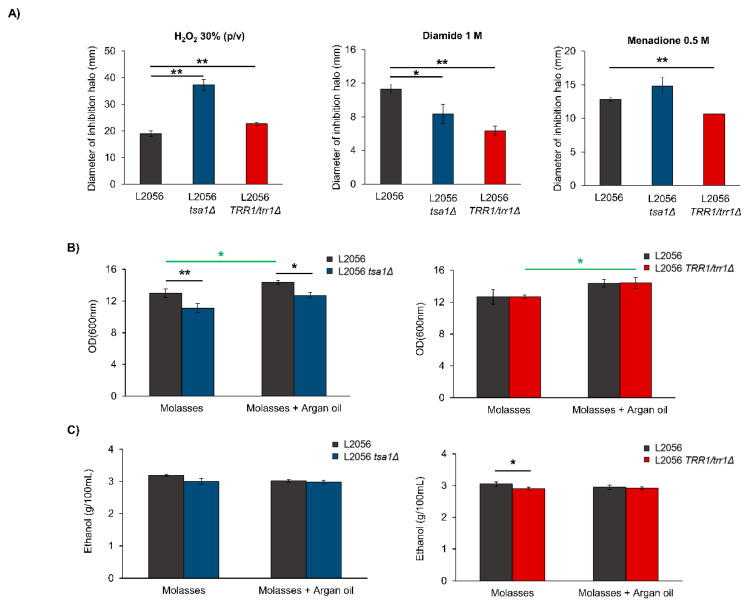
The *tsa1*Δ mutant phenotype. (**A**) Oxidative stress analysis of the L2056 *tsa1*Δ and L2056 *trr1*Δ/*TRR1* mutants. The diameter of the inhibition halo caused by the indicated amount of oxidant molecule is shown. (**B**) Growth measured by OD600 of the wild type, L2056 *tsa1*Δ and L2056 *trr1*Δ/*TRR1* mutants after 24 h in flasks with molasses, with and without argan oil 12 µL/mL. (**C**) Ethanol produced during the described experiment. Experiments were carried out in triplicate. The mean and standard deviation are shown (* *p* < 0.05; ** *p* < 0.01, Student’s *t* test).

The previous results indicated that peroxiredoxin *TSA1* was the most relevant one in growth terms; thus, both copies of the *TSA1* gene were deleted from commercial diploid wine strain L2056, the parental strain of strain C9, to test this deletion in a genetic background closer to industrial conditions. As a gene further up in the thioredoxin system, *TRR1* deletion in such a strain was performed. After knocking out a first copy, deleting the second one was unsuccessful. In a previous work, we used a *trr1*Δ mutant in haploid strain C9 [15], but thioredoxin reductase deletion was indicated to possibly be deleterious in a genetic background-dependent way. In some laboratory strains, that mutation has been described as lethal [40], as indicated in the *Saccharomyces* Genome Database. Heterozygous strain L2056 *trr1*/*TRR1* was tested against different oxidative insults together with *TSA1* deletion (Figure 2A). The mutant happens to be more sensitive to the presence of hydrogen peroxide.*tsa1*Δ mutant, is very sensitive to this oxidant, as expected. Therefore, full thioredoxin reductase activity is required for peroxiredoxins to detoxify H_2_O_2_, and the heterozygotic mutant is useful for assessing a reduction in thioredoxin reductase. However, the mutants showed increased tolerance to diamide (a thiol group oxidant). This could imply that alternative redox mechanisms, such as glutathione synthesis or superoxide dismutase, could be active to compensate for the loss of thioredoxin reductase and peroxiredoxin Tsa1. Superoxide generator menadione gives a complex phenotype, as the heterozygotic mutant is more tolerant to it, but *TSA1* deletion gives more sensitivity. Therefore, Trr1 may be controlling compensatory mechanisms that may act through other peroxiredoxins.

Therefore, the two mutants defective in key thioredoxin-peroxiredoxin system steps in a commercial wine strain were tested under conditions mimicking industrial processes and industrial growth media. First, mutants were grown in flasks with shaking in sugar beet molasses. This setup allowed a direct comparison to be made between the mutants and changes in media composition, such as the effect of a food-grade antioxidant such as argan oil, which was also tested for that condition. Growth was estimated by measuring OD_600_ after 24 h growth, a time when all tested industrial *S. cerevisiae* strains consume completely the sucrose present in the medium [34]. Growth in the *tsa1*Δ mutant was impaired; thus, this peroxiredoxin was necessary for cell proliferation in a different growth medium and a distinct genetic background (Figure 2B). Adding argan oil significantly increased growth, as expected from a previous work conducted with T73 strain [8], due to its protective effect. However, even in the presence of that antioxidant, *tsa1*Δ mutant displayed a growth defect; thus, its lack of function could not be fulfilled by any chemical antioxidant under this condition. Heterozygous mutant *TRR1*/*trr1*Δ presented no growth defect as compared with the reference strain. Thus, lower thioredoxin reductase activity sufficed to fulfill all the growth requirements of this strain. Adding argan oil increased the final density of this mutant, which means that certain oxidative damage in the mutant is prevented by adding argan oil. Despite the differences in growth, no significant changes were found in the amount of ethanol in the *TSA1* deletion mutant, but *TRR1* reduction slightly decreased it in the absence of argan oil (Figure 2C).

### 3.3. Peroxiredoxin TSA1 Plays a Major Role in Redox Balance during Growth on Molasses

**Figure 3:**
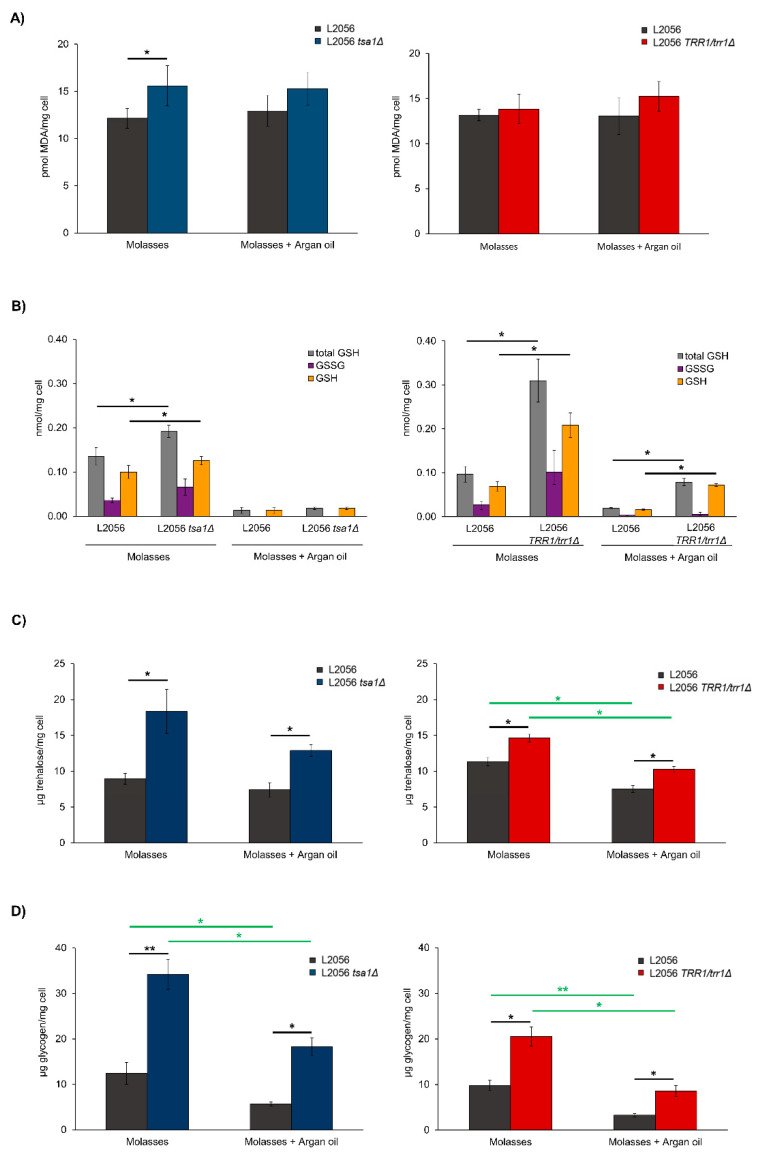
Oxidative stress parameters during growth in molasses. L2056, the L2056 *tsa1*Δ mutant and L2056 *trr1*Δ/*TRR1* were grown for 24 h in flasks with molasses, with and without argan oil 12 μL/mL. (**A**) Lipid peroxidation. (**B**) Total, reduced and oxidized glutathione. (**C**) Accumulated trehalose. (**D**) Accumulated glycogen. Experiments were carried out in triplicate. The mean and standard deviation are shown (* *p* < 0.05; ** *p* < 0.01, Student’s *t* test).

Next, a series of oxidative damage or unbalance markers were tested for the above-mentioned mutants after growth on molasses. The easiest way to assess oxidative damage is to measure lipid peroxidation through the malondialdehyde levels (Figure 3A). This parameter was high in the *TSA1* deletion strains compared to the parental strain, indicating that peroxiredoxin is required for preventing oxidative damage during growth in molasses. This difference reduced in the presence of argan oil and became insignificant. Therefore, argan oil possesses an in vivo antioxidant function that prevents biological damage during biomass propagation, but only when cellular antioxidant defenses do not fully function. As the heterozygous *TRR1*/*trr1*Δ strain did not show that oxidative damage signature, the increase in mortality when challenged with peroxides is not caused by lipid peroxidation, and argan oil treatment did not change this pattern.

A key player in redox control and good indicator of the antioxidant response status is tripeptide glutathione. The mutation of *TSA1* globally increased total glutathione (Figure 3B). This could be caused by compensatory mechanisms; if the main peroxiredoxin is missing, glutathione may increase to fuel glutathione-dependent peroxidases. The same was true for reduced glutathione, which indicates that glutathione systems have excess capacity to play the roles of thioredoxin mechanisms. Oxidized glutathione was slightly higher in the mutant, which means that the oxidative damage caused by *TSA1* has accumulated. The same molecules were measured when argan oil was present in the growth medium. A global repression of all glutathione forms took place in both the wild type and mutant, suggesting the nonspecific down-regulation of glutathione metabolism when argan oil was present during growth. The oxidized version of glutathione was negligible when argan was added to growth media. A similar profile was seen for the heterozygous *TRR1* strain, but to a higher extent (Figure 3B). The total and reduced glutathione in the mutant strain significantly increased. Once again, argan oil lowered levels, but ratios were similar.

There are two sugar-based molecules relevant for stress tolerance in yeast: trehalose and glycogen. Trehalose was measured after growth in molasses as described (Figure 3C). Trehalose content increased, and almost doubled, in the *tsa1*Δ mutant. The effect of argan oil was surprisingly negative on trehalose production, and in such a direction that it could indicate that both protection systems worked in opposite directions. Alternatively, the need of trehalose is lower when argan oil is present, as it provides a protective effect already. Even in the presence of argan oil, lack of *TSA1* rocketed trehalose content, which indicates that the role of *TSA1* is more determinant than argan oil as regards trehalose. The effect of deleting one copy of *TRR1* went in the same direction, but to a lesser extent: the mutant displayed increased trehalose accumulation, even in the presence of argan oil. Therefore, the thioredoxin system is required to repress trehalose synthesis, or trehalose bursts, to compensate for lack of peroxiredoxin activity. Glycogen is a reserve carbohydrate and, although it is produced under nutrient starvation, it is not considered an antistress molecule. The effect of the *tsa1*Δ mutant was, once again, a marked increase in glycogen regardless of argan oil being present or absent (Figure 3D). Interestingly, argan oil once again decreased glycogen accumulation. The heterozygous *TRR1*/*trr1*Δ strain showed increased glycogen, albeit not as high, but still resistant to the addition of argan oil.

### 3.4. TSA1 Impacts the Metabolic Transition from Fermentation to Respiration in Industrial Biomass Propagation Conditions

**Figure 4:**
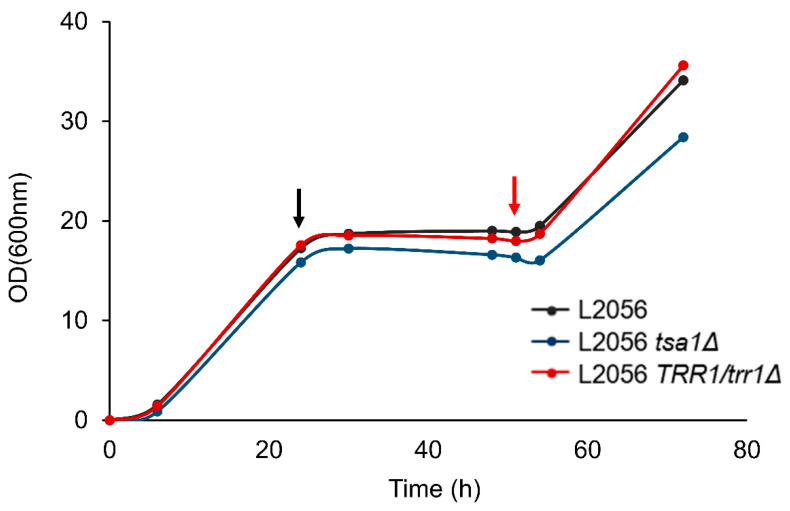
*TSA1* deletion impairs growth in bioreactor. Growth measured by OD_600_ of L2056, the L2056 *tsa1*Δ mutant and the L2056 *trr1*Δ/*TRR1* strains in fermenters by a batch/fed-batch approach. A black arrow indicates when sucrose is consumed. A red arrow denotes when the fed-batch is initiated.

Growth in flasks with molasses allowed many strains, mutants and media supplementations to be tested in parallel. However, to fully mimic the industrial process, a fermenter with coupled batch and fed-batch phases must be used [41]. We developed a synthetic molasses medium to make the process more reproducible, and to either enable the use of fluorescent dyes or help protein extraction (see below), which may be affected by industrial media impurities. Cells were first grown under batch conditions in such synthetic molasses with shaking. Under this condition, L2056 quickly grew and cells reached saturation and consumed all the sucrose in only 1 day (Figure 4). At 51 h, the produced ethanol was consumed and the fed batch was initiated, and cells resumed growth. The heterozygous mutant *TRR1*/*trr1*Δ showed a similar growth pattern to the parental strain, indicating that this enzymatic activity is not required at full power in these conditions. However, *TSA1* deletion mutant did not reach the same cellular density and remained lower when the fed-batch took place.

**Figure 5:**
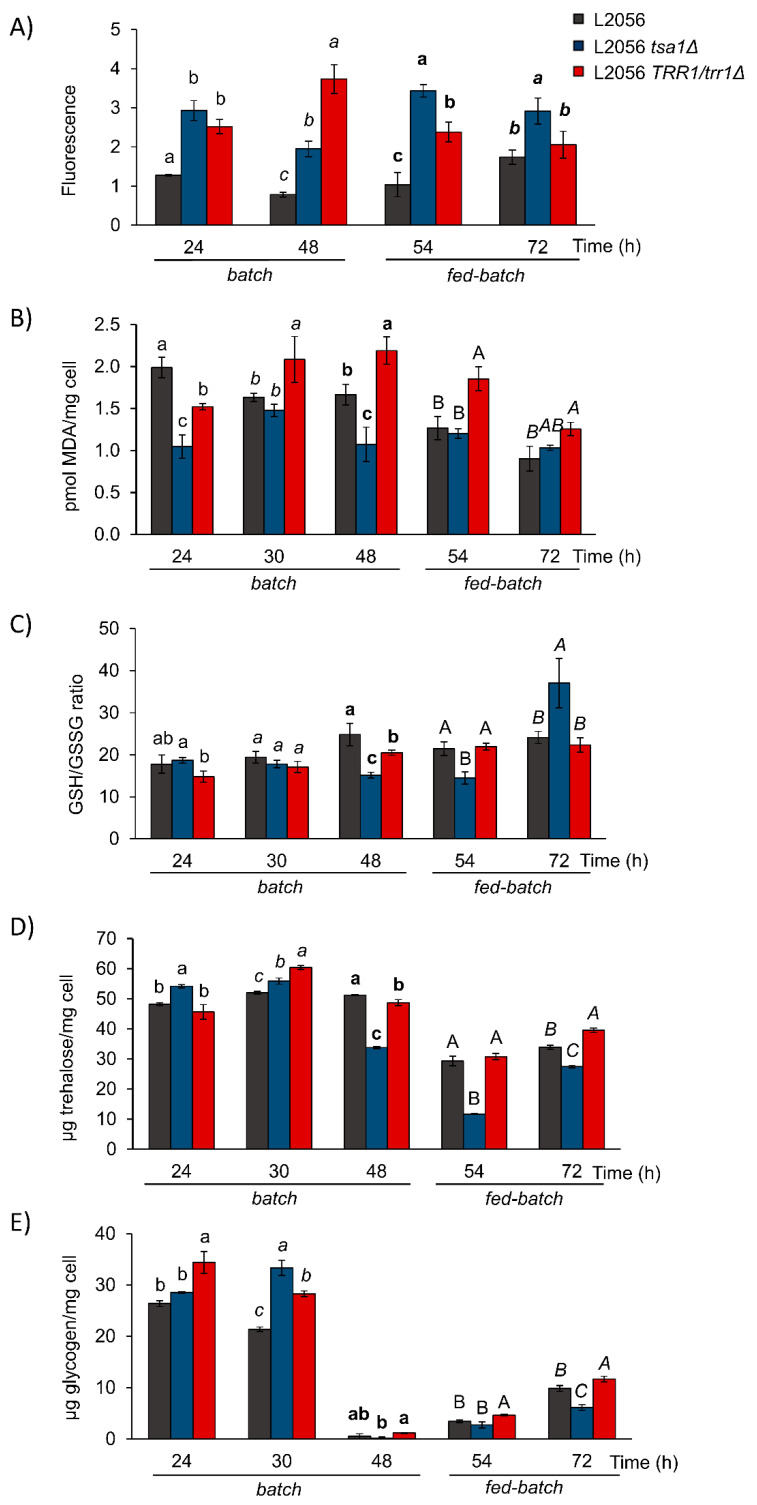
Oxidative stress parameters during growth in fermenters. L2056, the L2056 *tsa1*Δ mutant and L2056 *trr1*Δ/*TRR1* were grown as described in Figure 4. (**A**) ROS. DHE fluorescence is indicated. (**B**) Lipid peroxidation. (**C**) Glutathione GSH/GSSG ratio. (**D**) Trehalose. (**E**) Glycogen. Experiments were carried out in triplicate. The mean and standard deviation are shown. Means with the same letter are not significantly different (*p* < 0.05) at the same times according to Tukey’s test. Different font styles enable comparing the same strain in different times.

Next, some redox parameters were measured. Intracellular ROS were detected by the fluorescent dye DHE (Figure 5A). In the wild type, ROS remained mostly constant during the batch phase and increased with the fed-batch. With constant shaking, oxygen availability was good from the very beginning. *TSA1* deletion increased ROS at all the time points, which means that *TSA1* was relevant for oxidant detoxification under these growth conditions. Deletion of one copy of *TRR1* also increased ROS during batch, which indicates that the thioredoxin system is key for controlling redox status during growth in molasses in fermenters. The levels of lipid peroxidation, a mark of oxidative damage, slightly rose for longer times in the *TRR1*/*trr1*Δ mutant (Figure 5B), which was expected due to the diminished activity of the whole thioredoxin system. However, *TSA1* deletion did not fit this scheme because its deletion had no effect or peroxidation even decreased, which suggests that *TSA1* is not a key player in preventing lipid damage. The reduced/oxidized glutathione ratio indicated the status of the other redox controlling system (Figure 5C). The *tsa1*Δ mutant had a low ratio at the end of batch, which indicates that the redox balance tilted to a more oxidized state, which drags not only the thioredoxin system, but also the glutathione system. This was missing in the late fed-batch phase. The deletion of one copy of *TRR1* led to a mild effect or no effect at all; thus, partial thioredoxin reductase sufficed to control this parameter. Supplementary Figure S1 shows the amount of the total, reduced and oxidized glutathione. *TSA1* deletion increased total glutathione, which may indicate a compensatory mechanism. However, it went below the reference strain at the end of the batch, which suggests regulated interplay between both systems.

Trehalose and glycogen were measured under these conditions. As it occurs during growth in flasks, *TSA1* deletion brought about an increase in trehalose after 1 day (Figure 5D). However, for longer times, this mutation led to lower trehalose levels compared to the wild-type strain. Deletion of one copy of *TRR1* gave a more subtle phenotype, with minor variations that did not always move in the same way as *TSA1* deletion. The trehalose levels were similar throughout the process, but glycogen levels drastically dropped at the end of batch growth (Figure 5E), which indicated its mobilization when available nutrients were fully consumed. Deletion of either *TSA1* or one copy of *TRR*1 led to increased glycogen in the early batch culture. However, as the mutation did not impact glycogen consumption, the role of the thioredoxin system seems linked with biosynthesis, and not with glycogen catabolism.

### 3.5. TSA1 Does Not Play a Determinant Role during Vinification

**Figure 6:**
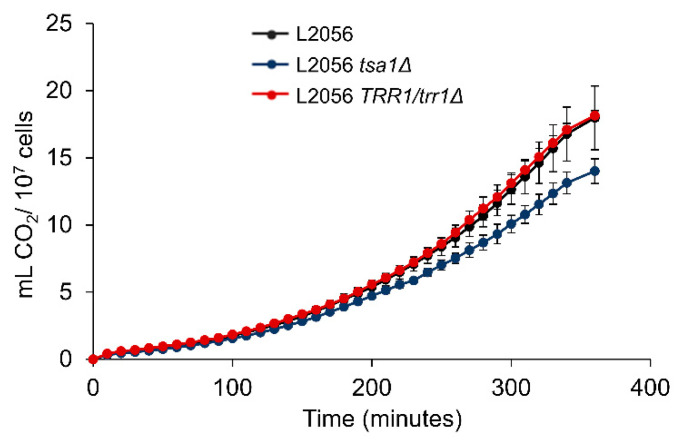
*TSA1* is required for full fermentative capacity. The capacity fermentative of L2056, the L2056 *tsa1*Δ mutant and the L2056 *trr1*Δ/*TRR1* strain in synthetic grape juice was measured by CO_2_ production. Experiments were carried out in triplicate. The mean and standard deviation are shown.

The next step was to analyze the impact on such mutations under winemaking conditions in order to better correlate the role of peroxiredoxin with metabolic cell status. First, the fermentative capacity of the strains of interest was measured in a cell growth-independent way. To do so, carbon dioxide production was measured in the cells inoculated in a sugar-rich medium; in this case, synthetic grape juice with 200 g/L of an equimolecular mix of glucose and fructose (Figure 6). Cells started producing CO_2_ very quickly, as they came from an overnight culture in YPD-rich medium, which means cells are metabolically active. Deletion of one copy of *TRR1* did not cause haploinsufficiency, and the mutant had full fermentative capacity. However, as *TSA1* deletion significantly reduced the fermentative capacity, it affects the central carbohydrate metabolism.

**Figure 7:**
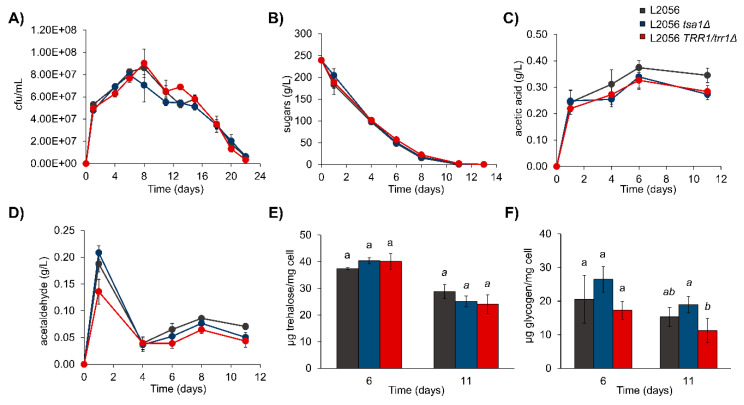
*TSA1* is not required for grape juice fermentation, but impacts acetic acid and acetaldehyde production. L2056, the L2056 *tsa1*Δ mutant and the L2056 *trr1*Δ/*TRR1* strains were grown in natural red grape juice. (**A**) Growth measured by CFU counting. (**B**) The fermentation profile measured by sugar consumption. (**C**) Acetic acid production during fermentation. (**D**) Acetaldehyde production during fermentation. (**E**) Trehalose at fermentation days 6 and 11. (**F**) Glycogen at fermentation days 6 and 11. Experiments were carried out in triplicate. The mean and standard deviation are shown. Means with the same letter are not significantly different (*p* < 0.05) at the same times according to Tukey’s test.

To analyze the impact of mutations on fermentative growth, the strains of interest were tested during red grape juice fermentation (Figure 7). Cell viability was followed by CFU counting (Figure 7A). The proliferation speed of the mutants under such conditions, the maximum cell density or the cellular death profile following showed no significant differences. This was a fermentative environment with a high monosaccharide concentration that could impose a different metabolic environment where thioredoxin-peroxiredoxin could be complemented by other systems. Moreover, metabolism, measured as reduced sugar consumption, did not reveal any differences (Figure 7B). *tsa1*Δ mutant showed a slight delay, which could reflect the diminished fermentative capacity, but it did not cause any further delay in vinification, which is completed normally, suggesting that the absence of the peroxiredoxin would be compensated in the long run. Metabolites were measured at the end of fermentation. No difference was found in the total ethanol amount (data not shown), which was expected for the same sugar consumption; additionally, no differences were found in final glycerol (data not shown), but acetic acid level was lower for both mutants. Thus, its profile was measured during fermentation (Figure 7C). Acetic acid built up for the first six fermentation days and it stabilized later. The initial synthesis rate of acetic acid was similar between strains, but in the mutants, it reached lower levels later in fermentation. Another metabolite of organoleptic abilities, linked with acetic acid, is acetaldehyde. The acetaldehyde profile differed (Figure 7D), with an initial burst taking place on the first fermentation day and stabilizing later during fermentation. A reduction in this early peak was observed with the *TRR1*/*trr1*Δ strain, and both mutants reached lower levels at the end of fermentation. Protective molecules, such as trehalose (Figure 7E) and glycogen (Figure 7F), were tested during grape juice fermentation when cells reached saturation (day 6), and also at the end of fermentation (day 11). Trehalose peaked when cells entered the stationary phase and decreased when sugars were depleted. No significant differences appeared in the mutant strains. Glycogen was, once again, higher halfway through fermentation than when it ended. Once more, there were no significant differences in the *TSA1* deletion strain for this parameter, although it tended to have slightly more glycogen. Therefore, the impact on protective carbohydrates during vinification does not seem to be strictly regulated by *TSA1*.

**Figure 8:**
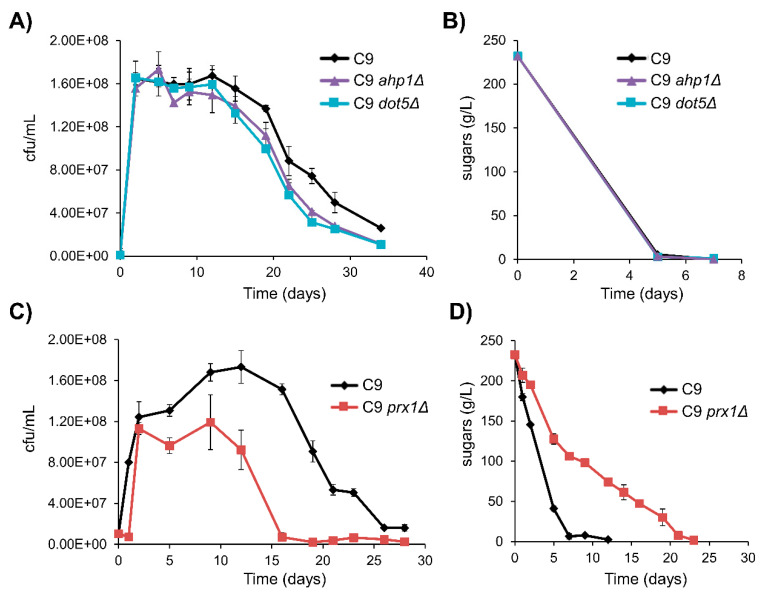
Mitochondrial peroxiredoxin plays a relevant role in fermentation. C9 and the derived mutants were grown in red grape juice. (**A**) CFUs of the C9 *ahp1*Δ and C9 *dot5*Δ mutants. (**B**) The reducing sugar consumption of this fermentation. (**C**) CFUs of the C9 *prx1*Δ and C9 reference strain. (**D**) The reducing sugar consumption of this fermentation. Experiments were carried out in triplicate. The mean and standard deviation are shown.

As *TSA1* plays no clear role in vinification, the deletion of other peroxidases in haploid strain C9 was tested during natural grape juice fermentation (Figure 8). Tsa2 played no role (data not shown). Alkylperoxidases Ahp1 (cytosolic) and Dot5 (nuclear) had no impact on growth, although cells tended to die at a faster rate (Figure 8A). No difference in sugar consumption was found (Figure 8B). However, mitochondrial peroxiredoxin mutation *prx1*Δ caused an increased lag phase, lower cell density and cells start dying faster. This scenario indicates that there is a problem once the stationary phase was reached (Figure 8C), which also caused defective sugar consumption (Figure 8D). This finding suggests a role of mitochondria in winemaking conditions.

## 4. Discussion

In recent yeasts, peroxiredoxin Tsa1 has emerged as a multifunctional protein with many activities other than its enzymatic ability to eliminate hydrogen peroxide [21]. Its dual function as a protein chaperone when oxidized is well-known [22], and this function is relevant for protein homeostasis under certain conditions such as zinc deficiency, which promotes misfolding [42]. These moonlighting abilities are conserved in other yeasts; for instance, *Candida albicans* Tsa1 has been linked with cell wall biogenesis [43]. As hydrogen peroxide can act as a second messenger at lower doses, *TSA1* has been proposed to act as a molecular sensor that can trigger apparently unrelated mechanisms such as light sensing [44] and circadian clocks [45].

Our aim was to understand the broader role of peroxiredoxins beyond stress tolerance in wine yeasts, and under conditions such as those that wine strains face during their industrial use; namely, from their biomass propagation in molasses to grape juice fermentation. From the simple growth experiments run following the growth curve in defined media with glucose and sucrose as carbon sources, it became clear that the main cytosolic peroxidase *TSA1* was the most relevant one, as its mutation caused impaired growth, even under conditions regarded as optimal for cell proliferation (Figure 1). However, this is no general growth defect per se: although a slight reduction in growth on molasses was observed both in flasks and fermenters (Figure 2 and Figure 4), fermentative growth in grape juice was not affected by *TSA1* deletion (Figure 7). Therefore, *TSA1* seems more relevant when transitions from fermentative to respiratory conditions happens, when more shaking is applied. Obviously, this enzyme is relevant for stress protection, and its mutation leads to an increase in ROS and a change in the glutathione ratio during growth in molasses, and in both flasks and fermenters (Figure 3 and Figure 5), which implies an impact on the cell redox status. However, there are changes in parameters that do not correlate easily with a mere redox-scavenging function. There is a complex pattern of regulation of protective carbohydrates, such as trehalose and glycogen, that do not correlate exactly with the anti-stress response. During growth in fermenter, *TSA1* has a negative role at the beginning of trehalose accumulation, but it is required to maintain its levels later on (Figure 5). A more direct regulation may be in place. *TSA1* has been recently described as a redox regulator of one of the main nutrient signaling pathways, protein kinase A. PKA is the main pathway controls trehalose and glycogen accumulation throughout the yeast life cycle [13]. *TSA1* plays no role in the protection conferred by argan oil during growth in molasses. Thus, other peroxiredoxin must be involved in this fact and further work needs to be done to clarify all these questions.

Lowering thioredoxin reductase activity by the deletion of one of the *TRR1* genes in a diploid wine strain leads to a similar phenotype for some, but not all, the phenotypes observed for *TSA1* deletion. Regulation of both trehalose and glycogen in flasks with molasses is shared, while growth defects only happen under some conditions. This could be due to the partial nature of the mutation, and indicates that no full reducing power is required for most events. However, given the moonlighting nature of Tsa1, some of its functions might not rely on peroxidase activity, which requires the whole thioredoxin system, but on its chaperone activity, which may act independently. The mutation in *TSA1* led to diminished fermentative capacity (Figure 6), which was shared with the mutation of both cytosolic thioredoxins *TRX1* and *TRX2* [19], and suggests that general glycolytic flux control might require the whole thioredoxin-peroxiredoxin system. In addition, we cannot rule out any unknown functions of *TSA1*. Tsa1 is known to physically interact with pyruvate kinase Cdc19 to inhibit its activity and enhance gluconeogenesis, with a non-peroxidase function as a redox-dependent target modulator [46]. A similar mechanism could take place for trehalose biosynthesis. A genetic interaction has been established between *TSA1* and the genes of the trehalose synthase complex *TPS1* and *TPS2* [24]. As no similar genetic interaction occurs with the trehalases that degrade trehalose, perhaps this metabolite’s levels are regulated at its synthesis, and not by regulating its degradation. No similar interaction occurs with the glycogen synthase enzyme. However, an alternative mechanism that could control carbohydrate metabolism may exist.

Although *TSA1* deletion did not disturb grape juice fermentation, it brought about the alteration of two interesting enological metabolites with a strong organoleptic impact: acetaldehyde and acetic acid (Figure 7). Although they can be metabolically ligated in a single redox reaction, their production profile during fermentation is different. Thus, the way that Tsa1 influences them could differ or be indirect. There are many alcohol and aldehyde dehydrogenases in the yeast genome that can be taken into account, but not one of them interacts genetically with *TSA1* [24]. Other peroxidases may play a different role under some growth conditions. Interestingly, mitochondrial peroxiredoxin Prx1 deletion has an impact during winemaking (Figure 8), while it is expendable in growth in other media (Figure 1). Therefore, further work has to be conducted to clarify the role of every peroxiredoxin for all growth conditions.

## Supplementary Materials

The following are available online at https://www.mdpi.com/2076-2607/8/10/1537/s1, Figure S1: Total, oxidized and reduced glutathione described in Figure 5. Table S1: Strains used in this work.
