# Supplementary material for: Wine Yeast Peroxiredoxin TSA1 Plays a Role in Growth, Stress Response and Trehalose Metabolism in Biomass Propagation
Source: Microorganisms. 2020 Oct 6;8(10):1537. doi: 10.3390/microorganisms8101537 (PMC7600145; doi:10.3390/microorganisms8101537)
Supplement: Supplementary file 1 [file microorganisms-08-01537-s001.pdf]

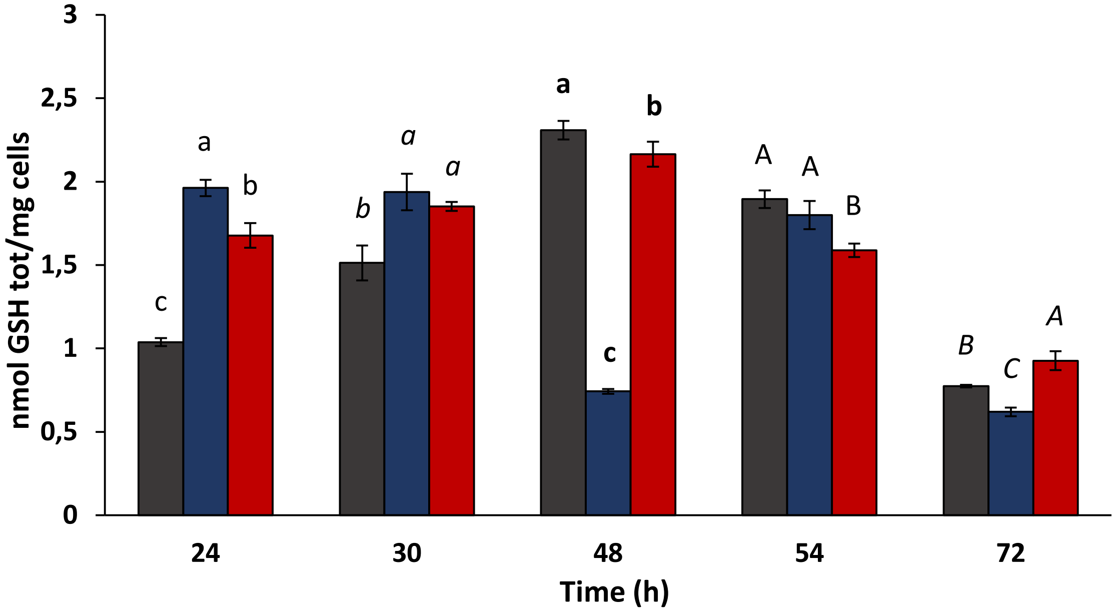

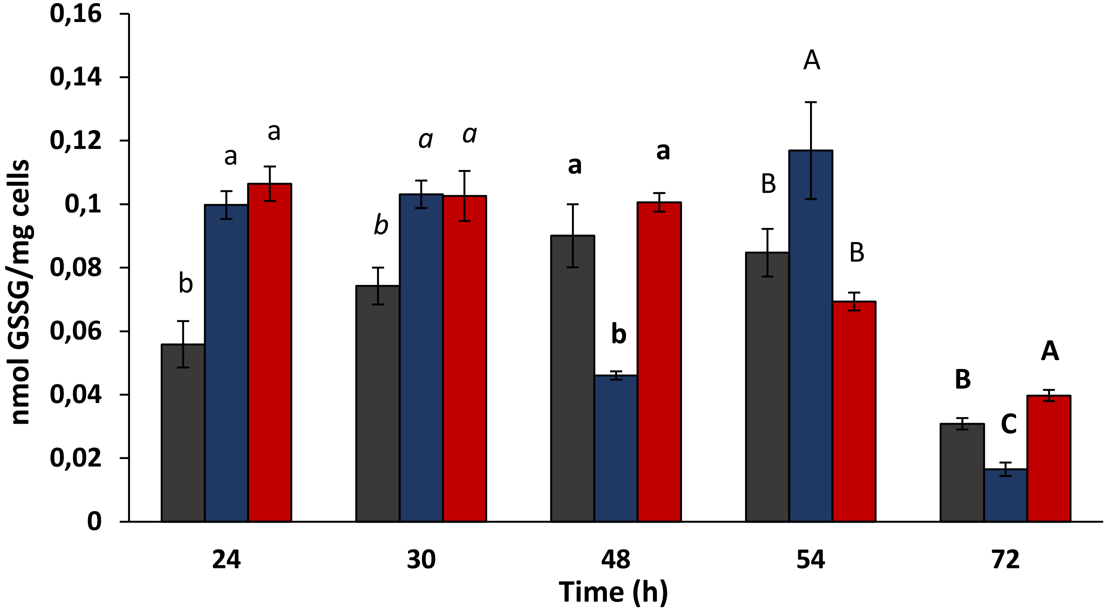

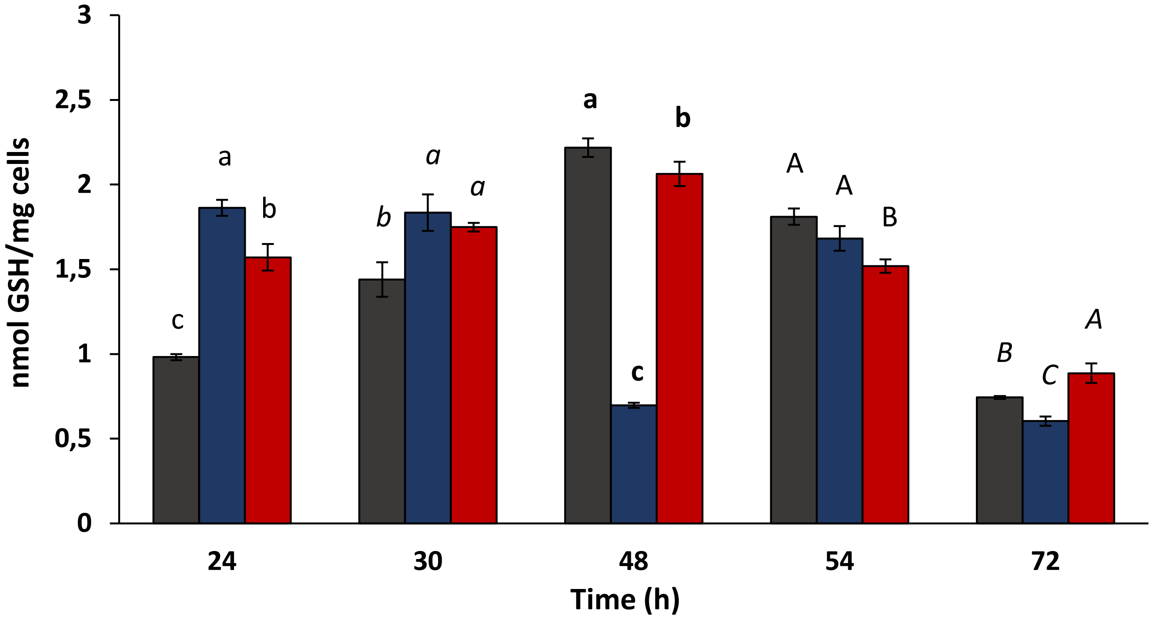

Figure S1: Total, oxidized and reduced glutathione described in Figure 5.

Supplementary Table S1: strains used in this work

**Strain**	**Genotype**	**Origin**
C9	C9 Mat a *ho::loxP*	Walker *et al.*, 2003
C9 *tsa1*	C9 *tsa1::loxP*	This work
C9 *tsa2*	C9 *tsa2::KanMX*	“ ”
C9 *dot5*	C9 *dot5::KanMX*	“ ”
C9 *ahp1*	C9 *ahp1:: KanMX*	“ ”
C9 *prx1*	C9 *prx1::KanMX*	“ ”
C9 *trx1*	C9 *trx1::KanMX*	Picazo et al., 2019
C9 *trx2*	C9 *trx2::KanMX*	Picazo et al., 2019
C9 *trx1 trx2*	C9 *trx1::KanMX trx2::loxP*	Picazo et al., 2019
C9 *trr1*	C9 *trr1::KanMX*	Picazo et al., 2018
L2056	Lalvin L2056	Lallemand
L2056 *tsa1*	L2056 *tsa1::KanMX tsa1::loxP*	This work
L2056 *trr1/TRR1*	L2056 *trr1::KanMX*	This work
